# Retraction: Small Language Models for Developing Agentic AI in Healthcare: A Comprehensive Systematic Review and Critical Analysis

**DOI:** 10.7759/cureus.r243

**Published:** 2026-09-09

**Authors:** Zain Khalpey, Nicholas King, Alyssa Abraham

**Affiliations:** 1 Department of Cardiothoracic Surgery, HonorHealth, Scottsdale, USA; 2 Department of Cardiology, HonorHealth, Scottsdale, USA; 3 Khalpey AI Lab, Department of Cardiothoracic Surgery, HonorHealth, Scottsdale, USA

This article has been retracted by the Editor-in-Chief. Following concerns raised by a reader, a post-publication review by the journal found that 25 of the 35 studies presented as included in this systematic review (Studies 11 through 35 in Tables 3 and 4, labeled "[Placeholder]") do not correspond to identifiable published sources, and that the characteristics attributed to several of the remaining included studies do not match the cited publications. The authors were unable to identify the studies in question. The authors confirmed the factual findings set out above and proposed corrections. They disagree with the decision to retract rather than correct the articles.

